# Supplementary material for: Ultrastructural visualization of 3D chromatin folding using volume electron microscopy and DNA in situ hybridization
Source: Nat Commun. 2020 May 1;11:2120. doi: 10.1038/s41467-020-15987-2 (PMC7195386; doi:10.1038/s41467-020-15987-2)
Supplement: Supplementary file 6 — Description of Additional Supplementary Files [file 41467_2020_15987_MOESM6_ESM.pdf]

Title: SUPPLEMENTARY MOVIE 1.
Description: Rotating structure, movie file for sID 50

Title: SUPPLEMENTARY MOVIE 2.
Description: Rotating structure, movie file for sID 12

Title: SUPPLEMENTARY MOVIE 3.
Description: Rotating structure, movie file for sID 42
