# Supplementary material for: Ultrastructural visualization of 3D chromatin folding using volume electron microscopy and DNA in situ hybridization
Source: Nat Commun. 2020 May 1;11:2120. doi: 10.1038/s41467-020-15987-2 (PMC7195386; doi:10.1038/s41467-020-15987-2)
Supplement: Supplementary file 7 — Reporting Summary [file 41467_2020_15987_MOESM7_ESM.pdf]

nature research

Corresponding author(s): Grzegorz M. Wilczynski

Last updated by author(s): Mar 25, 2020

Reporting Summary

Nature Research wishes to improve the reproducibility of the work that we publish. This form provides structure for consistency and transparency in reporting. For further information on Nature Research policies, see Authors & Referees and the Editorial Policy Checklist .

Statistics

For all statistical analyses, confirm that the following items are present in the figure legend, table legend, main text, or Methods section.

n/a	Confirmed	
☐	☒	The exact sample size (*n*) for each experimental group/condition, given as a discrete number and unit of measurement
☐	☒	A statement on whether measurements were taken from distinct samples or whether the same sample was measured repeatedly
☐	☒	The statistical test(s) used AND whether they are one- or two-sided<br>*Only common tests should be described solely by name; describe more complex techniques in the Methods section.*
☒	☐	A description of all covariates tested
☐	☒	A description of any assumptions or corrections, such as tests of normality and adjustment for multiple comparisons
☐	☒	A full description of the statistical parameters including central tendency (e.g. means) or other basic estimates (e.g. regression coefficient) AND variation (e.g. standard deviation) or associated estimates of uncertainty (e.g. confidence intervals)
☐	☒	For null hypothesis testing, the test statistic (e.g. *F*, *t*, *r*) with confidence intervals, effect sizes, degrees of freedom and *P* value noted<br>*Give P values as exact values whenever suitable.*
☒	☐	For Bayesian analysis, information on the choice of priors and Markov chain Monte Carlo settings
☒	☐	For hierarchical and complex designs, identification of the appropriate level for tests and full reporting of outcomes
☒	☐	Estimates of effect sizes (e.g. Cohen's *d*, Pearson's *r*), indicating how they were calculated

Our web collection on statistics for biologists contains articles on many of the points above.

Software and code

Policy information about availability of computer code

Data collection	For the data collection we used Fiji distribution of ImageJ (https://imagej.net/Fiji/Downloads#Life-Line_Fiji)_and Gatan Microscopy Suite Software (commercially available). The images of nuclei were cropped manually, 3D visualizations (Fig. 1,2 and Supplementary Figure 3) were prepared using Amira (FEI), PartSeg (https://4dnucleome.cent.uw.edu.pl/PartSeg/) and UCSF Chimera software.
Data analysis	For data statistical analysis we used SPPSS software; for image reconstructions we used Partseq program (freely available at: https://4dnucleome.cent.uw.edu.pl/PartSeg/), Amira (ThermoFisher), Imaris (Bitplane) and Chimera (UCSF). For the defining subdomains within the 3D structures found, we utilized our custom made Python scripts, which are provided together with the manuscript, available from Github repository, https://github.com/3DEMISH/3D-EMISH.

For manuscripts utilizing custom algorithms or software that are central to the research but not yet described in published literature, software must be made available to editors/reviewers. We strongly encourage code deposition in a community repository (e.g. GitHub). See the Nature Research guidelines for submitting code & software for further information.

Data

Policy information about availability of data

All manuscripts must include a data availability statement. This statement should provide the following information, where applicable:

- Accession codes, unique identifiers, or web links for publicly available datasets
- A list of figures that have associated raw data
- A description of any restrictions on data availability

3D-EMISH image processing code and data files are available at the following public 456 repository server: https://github.com/3DEMISH/3D-EMISH

Field-specific reporting

Please select the one below that is the best fit for your research. If you are not sure, read the appropriate sections before making your selection.

☒ Life sciences ☐ Behavioural & social sciences ☐ Ecological, evolutionary & environmental sciences

For a reference copy of the document with all sections, see nature.com/documents/nr-reporting-summary-flat.pdf

Life sciences study design

All studies must disclose on these points even when the disclosure is negative.

Sample size	This is a descriptive study, therefore no comparison (control vs. experimental group) was performed. In choosing the number of structures for morphological-statistical analysis we relied on our experience from previous studies [Ito et al., Loss of neuronal 3D chromatin organization causes transcriptional and behavioural deficits related to serotonergic dysfunction, Nat Commun. 2014 Jul 18;5:4450. doi: 10.1038/] and [Walczak et al., Novel higher-order epigenetic regulation of the Bdnf gene upon seizures, J Neurosci. 2013 Feb 6;33(6):2507-11. doi: 10.1523/JNEUROSCI.1085-12.2013]. On the basis of the aforementioned studies, we estimated a sufficient number of cellular nuclei that allowed for quantitative analysis of nuclear sructure changes, by which we expected that a similar statistical sample is needed for chomatin structure characterization. The particular statistical sample size was restriced from the top by the capacity of the imaging system.
Data exclusions	Some of the images were excluded after visual inspection that showed defects related to the imperfect cutting of the sample by the diamond knife
Replication	There were two biological replicates in the study. All attempts were succsessful.
Randomization	There was no treatment group, therefore a randomization was not relevant to our study.
Blinding	Blinding was not relevant to our study, because of its descriptive rather than comparative nature.

Reporting for specific materials, systems and methods

We require information from authors about some types of materials, experimental systems and methods used in many studies. Here, indicate whether each material, system or method listed is relevant to your study. If you are not sure if a list item applies to your research, read the appropriate section before selecting a response.

Materials & experimental systems

n/a	Involved in the study	
☐	☒	Antibodies
☐	☒	Eukaryotic cell lines
☒	☐	Palaeontology
☒	☐	Animals and other organisms
☒	☐	Human research participants
☒	☐	Clinical data

Methods

n/a	Involved in the study	
☒	☐	ChIP-seq
☒	☐	Flow cytometry
☒	☐	MRI-based neuroimaging

Antibodies

Antibodies used	1. Anti-Avidin-FITC antibody, Mouse (Sigma; F1296, diltution 1:100; final concentration: 20 µg/ml).<br><br>2. Anti-Digoxigenin-Rhodamine, Sheep (Roche;11207750910) labelled probe with anti-digoxigenin-Alexa Fluor 647 (Jackson ImmunoResearch; diltution 1:250; final concentration: 2 µg/ml).<br><br>3. Anti-Sheep-Rhodamine (TRITC) (Jackson ImmunoResearch 713-025-003; diltution 1:100; final concentration: 10 µg/ml)). DNA was counterstained using Hoechst 33342 (Molecular Probes), and samples were mounted with Vectashield (Vector Laboratories).<br><br>4. Anti-Digoxigenin-Alexa Fluor 647 (Jackson ImmunoResearch 200-602-156; diltution 1:250; final concentration: 2 µg/ml).
Validation	1. Anti-Avidin–FITC antibody, Mouse (Sigma; F1269): validated among others in sections of human tonsils by Sigma and rat neurons in detection of DNA fluorescence in situ hybridization (FISH) signals in our laboratory (Walczak et al., Journal of Neuroscience 2013), Validated also in this manuscript in human GM12878 cell line based on the assumption that FISH gives two or four specific signals (Fig. 2B).<br>2. Anti-Digoxigenin-Rhodamine, Sheep (Roche; 11207750910) : validated by multiple investigators in sections and cell lines derived from many species in detection of FISH signals. Validated also in this manuscript in human GM12878 cell line based on the assumption that FISH gives two or four specific signals (Fig. 2B).

	3. Anti-Digoxigenin-Alexa Fluor 647, Mouse (Jackson ImmunoResearch; 200-602-156): validated among others in human cell lines in detection of FISH signals (Demaerel et al., Genome Res. 2019). Validated also in our laboratory in human GM12878 cell line using confocal microscopy (based on the assumption that FISH gives two or four specific signals) and iPALM (Supplementary Fig. 4).

Eukaryotic cell lines

Policy information about cell lines

Cell line source(s)	GM12878 (human lymphoblastoid) cells were purchased from commercial cell line, GM12878, Coriell Institute.
Authentication	The cells authentication was done by the Jackson Laboratory for the Genomic Medicine according to ATCC (American Type Culture Collection) recommended authentication tests (https://www.atcc.org/en/Services/Testing_Services/Cell_Authentication_Testing_Service/Cell_Line_Authentication_Test_Recommendations.aspx): Morphology check by optical observation of a magnified cell culture and Growth curve analysis. Any cells showing inconsistent growth properties were discarded.
Mycoplasma contamination	The cell line was tested negative for mycoplasma contamination.
Commonly misidentified lines (See ICLAC register)	No commonly misidentified cell lines were used in the study.
